# Performance of Segmented Thermoelectric Cooler Micro-Elements with Different Geometric Shapes and Temperature-Dependent Properties

**DOI:** 10.3390/e20020118

**Published:** 2018-02-11

**Authors:** Carlos Alberto Badillo-Ruiz, Miguel Angel Olivares-Robles, Pablo Eduardo Ruiz-Ortega

**Affiliations:** Instituto Politecnico Nacional, Coyoacan 04430, Mexico

**Keywords:** thermoelectric, microcooler, Thomson effect, segmented

## Abstract

In this work, the influences of the Thomson effect and the geometry of the p-type segmented leg on the performance of a segmented thermoelectric microcooler (STEMC) were examined. The effects of geometry and the material configuration of the p-type segmented leg on the cooling power ($Q_{c}$) and coefficient of performance (COP) were investigated. The influence of the cross-sectional area ratio of the two joined segments on the device performance was also evaluated. We analyzed a one-dimensional p-type segmented leg model composed of two different semiconductor materials, $Bi_{2}Te_{3}$ and ${\left(Bi_{0.5}Sb_{0.5}\right)}_{2}Te_{3}$. Considering the three most common p-type leg geometries, we studied both single-material systems (using the same material for both segments) and segmented systems (using different materials for each segment). The COP, $Q_{c}$ and temperature profile were evaluated for each of the modeled geometric configurations under a fixed temperature gradient of ΔT = 30 K. The performances of the STEMC were evaluated using two models, namely the constant-properties material (CPM) and temperature-dependent properties material (TDPM) models, considering the thermal conductivity (κ(T)), electrical conductivity (σ(T)) and Seebeck coefficient (α(T)). We considered the influence of the Thomson effect on COP and $Q_{c}$ using the TDPM model. The results revealed the optimal material configurations for use in each segment of the p-type leg. According to the proposed geometric models, the optimal leg geometry and electrical current for maximum performance were determined. After consideration of the Thomson effect, the STEMC system was found to deliver a maximum cooling power that was 5.10% higher than that of the single-material system. The results showed that the inverse system (where the material with a higher Seebeck coefficient is used for the first segment) delivered a higher performance than the direct system, with improvements in the COP and $Q_{c}$ of 6.67% and 29.25%, respectively. Finally, analysis of the relationship between the areas of the STEMC segments demonstrated that increasing the cross-sectional area in the second segment led to improvements in the COP and $Q_{c}$ of 16.67% and 8.03%, respectively.

## 1. Introduction

Thermoelectric microcoolers (TEMCs), also known as solid-state cooling devices, offer a variety of advantages such as not generating vibrations, not requiring the use of refrigerants and not needing constant maintenance. These devices also provide clean energy without the emission of harmful gases into the environment and thus represent a valuable alternative in certain industrial processes that require cooling control. The most important field of application for segmented thermoelectric devices is energy generation, owing to the capacity of these devices to work at high temperatures (up to 1200 K). One remarkable application is the radioisotope thermoelectric generator used in the Voyager probes; another is the utilization of high-temperature exhaust gases from diesel engines as a power source [1,2]. However, the greatest problem associated with thermoelectric cooler (TEC) devices is their low system performance, which is fundamentally limited by the material properties, i.e., Seebeck coefficient (α), electrical conductivity (σ) and thermal conductivity (κ), regardless of the design or configuration of the materials in the segmented TEC [3]. Segmented TECs are systems composed of two or more distinct semiconductor materials in the p-type and n-type legs. Thermoelectric devices such as microcoolers are influenced by four effects: the Seebeck effect, Peltier effect, Joule effect and Thomson effect. Among these, the Thomson effect has been neglected in several studies owing to its small magnitude compared with the other effects and the fact that a good approximation can be obtained using ideal equations (which do not consider the Thomson effect) [4,5]. The Thomson effect relates to the heat absorbed or released, depending on the direction of the electrical current flow, along a semiconductor material possessing a temperature gradient [6]. Thomson heat has been shown to play an important role in increasing or decreasing the performance of thermoelectric systems [7]. Thus, by considering a segmented thermoelectric microcooler (STEMC) system based on temperature-dependent material properties (TDPMs), we are able to determine the influence of the Thomson effect on a STEMC. Previous research has proposed a design methodology based on computational and analytical modeling, which was used to derive the optimum efficiency and geometry of segmented $Bi_{2}Te_{3}$–PbTe thermoelectric generators (TEGs) and led to a peak efficiency of 5.29% for ΔT of 324.6 K [8]. Yoon and co-workers have previously shown that segmented thermoelectric materials can offer improved efficiency over a wide temperature range and, with an optimization of the length of each segment when a diffusion layer is considered, with proper control, can increase the efficiency of the thermoelectric material for a large temperature gradient [9]. The Thomson effect in these systems was also studied by Lamba and Kaushik in their thermodynamic analysis of an exoreversible thermoelectric generator, including the influence of the Thomson effect and the leg geometry on the power output and efficiency [10]. Fabián-Mijangos and colleagues developed and experimentally validated a novel design for a thermoelectric module possessing asymmetrical legs with a truncated square pyramid shape, and the results demonstrated that the geometric configuration of the device legs significantly improved the thermoelectric performance of the device [11]. As the previously-mentioned works considered segmented TEGs rather than TECs, the goal of this work was to investigate whether similar performance improvements could be achieved using segmented TECs. Consequently, the influence of the geometries of the semiconductor elements in the thermoelectric devices was studied in an effort to improve the device efficiency. The current paper deals with the formulation of parameters to describe the characteristics and performance of thermoelectric cooling devices, because these thermoelectric systems are of current interest, as well as how to obtain improvements in the coefficient of performance COP [12]. As mentioned above, the performance of TEG devices can be improved by the segmentation of the semiconductor elements, so similar results may be expected for segmented thermoelectric microcoolers. These systems have been previously investigated; for example, De Aloysio and co-workers studied the ultrafast thermal behavior of a micro-thermoelectric cooler under the hyperbolic heat conduction model and demonstrated an increase in the COP of approximately 27% [13]. Cai and co-workers also recently presented a novel operating mode of the thermoelectric module for electronic cooling devices, mainly focusing on the effects of the thermoelectric properties [14]. A very recent work by Su and colleagues demonstrated that thermoelectric micro-refrigerators represent an attractive solid-state solution for on-chip thermal management of microelectronics owing to their unique advantages and proposed a free-standing planar design for a high-performance thermoelectric micro-refrigerator based on thin-film technology taking into account electrical contact resistances [15]. A similar attempt to optimize TECs according to geometric parameters was reported by Lv and co-workers, who proposed a new TEC design with a variable semiconductor cross-sectional area to improve the transient supercooling characteristics [16]. These investigations are important to meet the requirements of new applications where improved ways to cool systems are needed, such as central processing unit (CPU) cooling. Liu and co-workers proposed a thermoelectric mini cooler coupled with a micro-thermosiphon to serve as a CPU cooling system, and the experimental results indicated that the cooling performance was improved with increasing thermoelectric operating voltage [17]. Multistage thermoelectric cooler systems, which are devices formed by two or more simple stages connected electrically and thermally in series and designed to provide significantly higher temperature differentials than are obtainable with standard single-stage modules, also offer great potential for realizing higher performance coefficients, and these systems are currently being developed using various manufacturing techniques [18]. Karimi and co-workers used a 5 mm${}^{2}$ surface area, and the cold temperature was set at 278 K; the multistage thermoelectric cooler dissipates heat through a forced convection heat sink to ambient air at 298 K; the results shows that a maximum of 55 W cm${}^{-2}$ is achieved using an electric current I=8 A [19]. Sharma and co-workers worked with a hot temperature that remains constant at 303 K, and the cold temperature varies from 253–273 K (the heat is rejected at constant temperature at 300 K); their results show that a maximum COP of 1.48 with a rate of refrigeration of 0.29 can be achieved [20]. In Wang’s works, using operating conditions of hot and cold temperature of 300 K and I=8 A is probed, and the highest cooling capacity of 1.475 W and COP of 0.374 can be achieved [21]. Cheng and co-workers studied a method based on the genetic algorithm where the cold side temperature of the colder stage was set to 210 K and the hot side temperature of the hotter stage was set to 300 K, finding that the maximum cooling capacity and the maximum COP for the first type two-stage TECs are of 0.73 W and of 0.019, respectively [22]. Therefore, research on segmented thermoelectric elements in one-stage systems is important to understand multi-stage systems with segmentation, that is using segmented semiconductor elements in each stage of the system, and to improve cooling power in thermoelectric devices. In our study, various thermoelectric segmented p-type micro-elements and their configurations using different semiconductor materials are studied using a one-dimensional model of a segmented thermoelectric microcooler (STEMC), composed of two different semiconductor materials, ${\left(Bi_{0.5}Sb_{0.5}\right)}_{2}Te_{3}$ and $Bi_{2}Te_{3}$, connected electrically and thermally in series with the temperature dependence of material properties, i.e., the Thomson heat is considered. Therefore, this work addresses the optimization of a TEMC system using different geometries and material configurations under a temperature difference imposed across the device. For these purposes, three legs of different geometric shapes and configurations are modeled.

The remainder of this paper is organized as follows. In Section 2, our model of a segmented TEMC is described in detail along with the corresponding heat balance equations and temperature in the junction ($T_{m}$), using the CPM and TDPM models. In Section 3, we describe the temperature dependences of the material properties, such as the Seebeck coefficient, thermal conductivity and electrical conductivity, that were considered in this paper. Our results are presented in Section 4, when the thermoelectric properties are considered to be either constant or temperature dependent, and the results obtained with both models are compared. The temperature profiles of the elements are also presented. Finally, the optimized leg geometry is presented according to the best cross-sectional area for maximum performance. In Section 5, we provide some pertinent concluding remarks.

## 2. One-Dimensional Model of a Segmented TEMC

**Figure 1:**
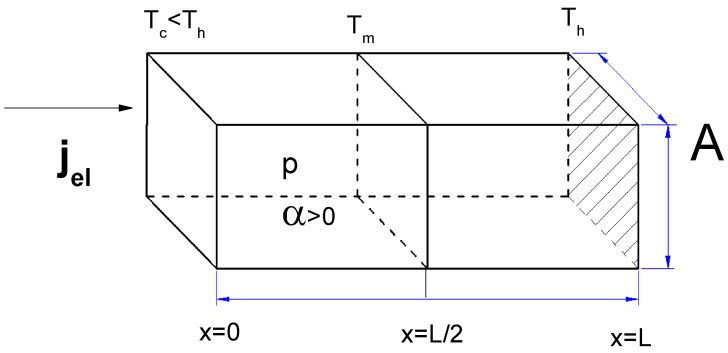
Factors considered in the 1D model of a segmented TEMC.

In this work, the thermoelectric effects in a segmented p-type Peltier element connected thermally and electrically in series are analyzed, as shown in Figure 1, where *A* is the cross-sectional area, *L* is the total length of the p-type element, $T_{c}$ and $T_{h}$ are the temperatures at the two ends of the element and $T_{m}$ is the temperature at the junction of the two segments.

In the framework of linear Onsager theory, the mutual interference of heat and electrical current flow in the thermoelectric process is considered in terms of the kinetic coefficients, which obey the Onsager reciprocity relations [23,24], and the related transport coefficients, such as the electrical conductivity (σ), thermal conductivity (κ) and Seebeck coefficient (α). Under isotropic conditions, a thermoelectric material is commonly considered based on linear constitutive equations for the electrical current density ($j_{el}$) and heat flux ($j_{q}$). It is well known that the spatial distribution of Peltier heat may exhibit singularities at the junctions of two different materials, which is of interest in this analysis because we consider a segmented p-type leg based on two different materials. Assuming steady-state conditions, the principles of the conservation of charge and energy lead to the following 1D governing differential equation [25,26]:

(1) $$\frac{\partial }{\partial x}\left(\kappa \frac{\partial T}{\partial x}\right)-j_{el}^{0}\frac{\partial (\alpha T)}{\partial x}+\alpha j_{el}^{0}\frac{\partial T}{\partial x}=-\frac{j{{}_{el}^{0}}^{2}}{\sigma }$$

where *T* is the temperature and $j_{el}^{0}$ is a constant electrical current density.

### 2.1. Temperature at the Junction ($T_{m}$)

The temperature at the junction ($T_{m}$) depends on the thermoelectric properties of the two segments composed of different materials. In fact, the junction temperature is involved in the calculation of both the COP and the cooling power ($Q_{c}$) of the system. To calculate the temperature at the junction ($T_{m}$), we must establish the boundary conditions for the temperatures $T_{c}$ and $T_{h}$ at the ends of the segments, where $T_{c}<T_{h}$.

If we consider both electrical current and temperature gradients within a Peltier device, then, in addition to Joule heat, Thomson heat must also be considered. A theoretical description of the Thomson effect begins with the assumption that the Seebeck coefficient (α(T)) depends on the temperature. From the Peltier coefficient Π(T)=Tα(T), we obtain the gradient:

(2) $$\nabla \Pi =\frac{d\Pi }{dT}\nabla T=\alpha \left(T\right)+T\frac{d\alpha (T)}{dT}\nabla T$$

The Thomson coefficient (τ) is related to the Peltier coefficient (Π(T))) and the Seebeck coefficient (α(T)) as follows:

(3) $$\tau =\frac{d\Pi }{dT}-\alpha \left(T\right)=T\frac{d}{dT}\frac{\Pi }{T}=T\frac{d\alpha (T)}{dT}$$

which leads to the equation:

(4) ∇Π=(τ+α)∇T

According to Equation (1), which describes temperature distribution, if we consider a model of temperature-dependent properties material (TDPM model), α(T), κ(T) and σ(T), temperature profiles T(x) can be calculated as follows:

(5) $$\kappa \left(T\right)\frac{\partial^{2}T}{\partial x^{2}}+\frac{d\kappa }{dT}{\left(\frac{\partial T}{\partial x}\right)}^{2}-j_{el}^{0}T\frac{d\alpha }{dT}\frac{\partial T}{\partial x}=-\frac{{j_{el}^{0}}^{2}}{\sigma (T)}$$

The 1D formulation of Equation (4) is given by:

(6) $$\frac{\partial \Pi }{\partial x}=\left(\tau +\alpha \right)\frac{\partial T}{\partial x}$$

Inserting the previous equation in Equation (1), we obtain the following differential equation including Thomson heat:

(7) $$\frac{\partial^{2}T}{\partial x^{2}}-\omega_{0i}\frac{\partial T}{\partial x}=-c_{0i}$$

with:

$$c_{0i}=\frac{j{{}_{el}^{0}}^{2}}{\sigma_{i}\kappa_{i}}\;\mathrm{and}\;\omega_{0i}=\frac{\tau_{i}}{\kappa_{i}}j{{}_{el}^{0}}^{2}.$$

where the subscript *i* is one or two as appropriate for Segment 1 or Segment 2, respectively. The solution to Equation (7) for the first segment of the p-type leg is:

(8) $$T{\left(x\right)}_{1}=c_{1}e^{\omega_{01}x}+c_{2}+\frac{c_{01}}{\omega_{01}}x$$

which represents the spatial temperature distribution in the first segment of our p-type leg model. The free constants $c_{1}$ and $c_{2}$ are determined by the boundary conditions of the thermoelectric problem. Then, for the first segment, we have the following boundary conditions:

(9) $$T\left(x=0\right)=T_{c},\;T\left(x=L/2\right)=T_{m}$$

The resulting constants are:

(10) $$c_{1}=-\frac{c_{01}L+2\omega_{01}\left(T_{c}-Tm\right)}{2\omega_{01}\left(e^{\frac{\omega_{01}L}{2}}-1\right)}\;c_{2}=T_{c}+\frac{c_{01}L+2\omega_{01}\left(T_{c}-Tm\right)}{2\omega_{01}\left(e^{\frac{\omega_{01}L}{2}}-1\right)}$$

The solution to Equation (7) for the second segment is:

(11) $$T{\left(x\right)}_{2}=c_{3}e^{\omega_{02}x}+c_{4}+\frac{c_{02}}{\omega_{02}}x$$

where $c_{3}$ and $c_{4}$ constants are determinate using the following boundary conditions:

(12) $$T\left(x=L/2\right)=T_{m},\;T\left(x=L\right)=T_{h}$$

The resulting constants are:

(13) $$c_{3}=-\frac{e^{-\frac{\omega_{02}L}{2}}\left[c_{02}L+2\omega_{02}\left(T_{m}-T_{h}\right)\right]}{2\omega_{02}\left(e^{\frac{\omega_{02}L}{2}}-1\right)}\;c_{4}=-\frac{c_{02}L-2\omega_{02}T_{m}}{2\omega_{02}}+\frac{c_{02}L+2\omega_{02}\left(T_{m}-T_{h}\right)}{2\omega_{02}\left(e^{\frac{\omega_{02}L}{2}}-1\right)}$$

The heat flux at the junction for the first segment in x=L/2 is given by:

(14) $$j_{q_{1}}\left(x=L/2\right)=-\kappa_{1}\left(\omega_{01}c_{1}e^{\omega_{01}x}+\frac{c_{01}}{\omega_{01}}\right)+j{}_{el}^{0}\alpha_{1}T_{m}$$

Similarly, for the second segment, we obtain:

(15) $$j_{q_{2}}\left(x=L/2\right)=-\kappa_{2}\left(\omega_{02}c_{2}e^{\omega_{02}x}+\frac{c_{02}}{\omega_{02}}\right)+j{}_{el}^{0}\alpha_{2}T_{m}$$

Now, solving for $T_{m}$, with $B=(e^{\frac{\omega_{01}L}{2}}-1)$ and $D=(e^{\frac{\omega_{02}L}{2}}-1)$, we obtain:

(16) $$T_{m}=\frac{2\kappa_{2}\omega_{01}c_{02}B\left(D-\frac{L\omega_{02}}{2}\right)-2\kappa_{1}\omega_{02}c_{01}BD\left(1-\frac{L\omega_{01}}{2}\right)+2\omega_{01}\left(e^{\frac{L\left(\omega 01+\omega 02\right)}{2}}\kappa_{1}T_{c}\omega_{01}+\kappa_{2}T_{h}\omega_{02}+e^{\frac{L\omega_{01}}{2}}\left(T_{h}\kappa_{2}\omega_{02}-T_{c}\kappa_{1}\omega_{01}\right)\right)}{2\omega_{01}\omega_{02}\left(BDj{{}_{el}^{0}}^{2}\left(\alpha_{2}-\alpha_{1}\right)+e^{\frac{L\left(\omega 01+\omega 02\right)}{2}}\kappa_{1}\omega_{01}-\kappa_{2}\omega_{02}+e^{\frac{L\omega_{01}}{2}}\left(\kappa_{2}\omega_{02}-\kappa_{1}\omega_{01}\right)\right)}$$

If we consider a constant material property (i.e., the CPM model), i.e., with constant fixed α, κ and σ, then ∂Π/∂x=α∂T/∂x and $\tau =T\frac{d\alpha }{dT}=0$. Thus, from Equation (1), we obtain:

(17) $$\frac{\partial^{2}T}{\partial x^{2}}=-c_{0i}\;\mathrm{with}\;c_{0i}=\frac{j{{}_{el}^{0}}^{2}}{\kappa_{i}\sigma_{i}}$$

The analytical solution of Equation (17) is:

(18) $$T{\left(x\right)}_{i}=-\frac{c_{0i}}{2}x^{2}+c_{1}x+c_{2}$$

Then, using the heat flux continuity condition at the junction between both stages, we solve for $T_{m}$ to obtain:

(19) $$T_{m}=\frac{\frac{2}{L}\left(\kappa_{2}T_{h}+\kappa_{1}T_{c}\right)+\frac{L}{4}\left(\kappa_{2}c_{02}+\kappa_{1}c_{01}\right)}{j{}_{el}^{0}\left(\alpha_{2}-\alpha_{1}\right)+\frac{2}{L}\left(\kappa_{1}+\kappa_{2}\right)}$$

### 2.2. Cooling Power ($Q_{c}$) and Coefficient of Performance (COP)

The Thomson effect has been studied together with the equation for thermal flux. According to the theory of non-equilibrium thermodynamics, the thermal flux can be calculated as:

(20) $$j_{q}\left(x\right)=-\kappa \frac{\partial T}{\partial x}+j_{el}^{0}\alpha T\left(x\right)$$

where $Q_{c}=j_{q}\left(0\right)A_{c}$ and the heat flow released ($Q_{h}=j_{q}\left(L\right)A$) can be calculated for the cold side (x=0) and the hot side (x=L), using the electrical current $I=j_{el}^{0}$ A. It is important to note that $Q_{c}$ and $Q_{h}$ are dependent on the cross-sectional area (*A*) and length (*L*), which allows the optimization to be performed based on geometric parameters. The COP of the system is determined by $Q_{c}$ and $Q_{h}$ as follows:

(21) $$COP=\frac{Q_{c}}{P_{el}}=\frac{Q_{c}}{Q_{h}-Q_{c}}$$

where $P_{el}$ is the electrical power input.

## 3. Materials with Temperature-Dependent Properties

To study the Thomson effect, in this work, we consider materials with temperature-dependent properties to represent the two thermoelectric materials used in the STEMC. The thermoelectric properties were determined based on experimental data using established equations for the $Bi_{2}Te_{3}$ material, and a polynomial adjustment was made for the ${\left(Bi_{0.5}Sb_{0.5}\right)}_{2}Te_{3}$ material [25,27,28]. Table 1 summarizes the temperature-dependent properties of the two p-type semiconductor materials.

**Figure 2:**
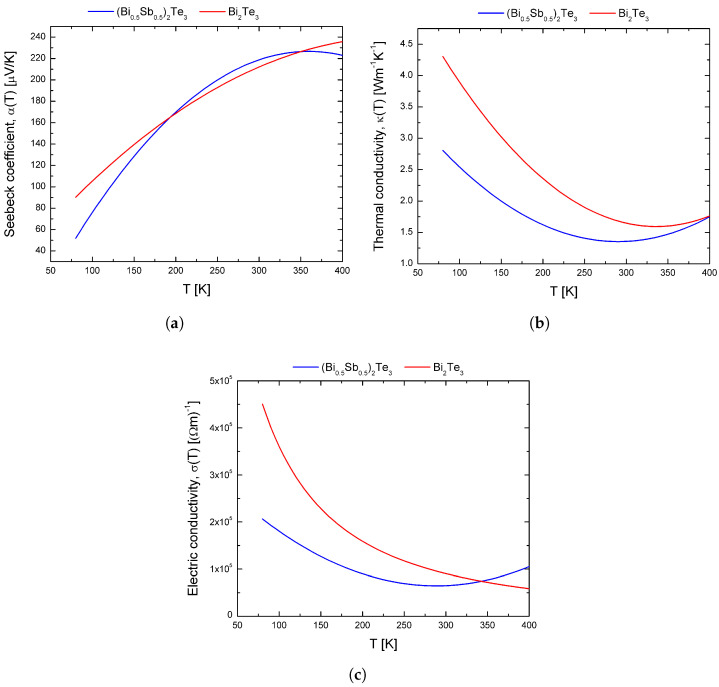
Polynomial approximation of experimental data: Temperature dependence of (**a**) Seebeck Coefficient, α(T), (**b**) Thermal Conductivity, κ(T) and (**c**) Electrical Conductivity, σ(T) (example data for a ${\left(Bi_{0.5}Sb_{0.5}\right)}_{2}Te_{3}$ and $Bi_{2}Te_{3}$ sample) [25,27].

Figure 2a–c shows the behavior of the properties of the materials in the temperature range of 80–400 K.

For the CPM model, the average values are used in the calculations, i.e., $\bar{\alpha }=\alpha \left(T_{avg}\right)$, $\bar{\sigma }=\sigma \left(T_{avg}\right)$ and $\bar{\kappa }=\kappa \left(T_{avg}\right)$ where $T_{avg}=\left(T_{c}+T_{h}\right)/2$. Alloys based on BiTe are the most commonly-preferred leg materials for applications using relatively small temperature gradients, and as such, they were considered in this work [29,30].

Previous studies have reported some strategies for improving the performance of thermoelectric devices. The results have demonstrated that thermoelectric power generation systems can be improved by modifying the geometry of the semiconductor elements and also by using two or more materials (i.e., segmentation) in the legs of a thermoelectric device. In other words, segmented thermoelectric generators typically possess a higher efficiency than those with homogeneous configurations. The performances of thermoelectric devices with various leg cross-sections have been evaluated, and the results demonstrated that devices with trapezoidal legs have a higher nominal power density than those with quadratic [31] or exponential variations in the leg cross-sections [32]. However, a similar analysis has not yet been conducted for thermoelectric cooling systems, and there is a lack of information in the literature concerning segmented legs in TECs. Therefore, we now present a procedure for the optimization of cooling systems based on the strategies mentioned above, which focuses on the optimization of a p-type segmented leg of a TEMC, including different leg geometries and material configurations. We analyze the influence of various parameters, such as the Thomson effect, material configuration and leg geometry, on the performance. In the following section, we analyze the performance of a segmented leg based on the materials shown in Table 1, as a function of the electrical current (*I*). The initial analysis revealed the performance of each material in the TEMC. Subsequently, and considering the Thomson effect, we compare the performances of a hybrid system with different materials in each segment of the p-type leg and a homogeneous system with the same material in both segments of the p-type leg. An analysis of the temperature distribution is also performed. Finally, we establish the optimum cross-sectional area ratio between the two segments to optimize the p-type leg system.

## 4. Results

**Figure 3:**
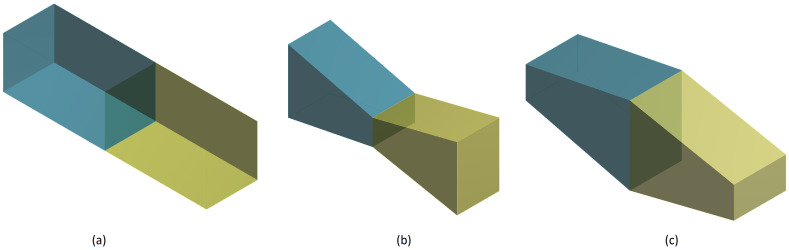
Segmented p-type leg shapes: (**a**) rectangular prism; (**b**) trapezoidal prism with a reduced junction contact area; and (**c**) trapezoidal prism with a large junction contact area.

The performance of TEC devices depends on many factors, such as the temperature gradient between the hot and cold ends of the leg, the material properties and the configuration and arrangement of the thermoelectric leg materials. Among these factors, leg geometry of the micro-elements and semiconductor materials configurations for segmented legs are crucial to be able to optimize a TEMC system. Therefore, we investigated the influence of the leg cross-sectional area on the cooling power. To achieve this objective, the three different leg geometry models depicted in Figure 3, namely a rectangular prism, a trapezoidal prism with a small junction contact area and a trapezoidal prism with a large junction contact area, were analyzed to predict their steady-state TEMC performance.

### 4.1. Optimum Material Configuration and Electric Current

In this section, we evaluate the optimum geometry for improving the performance of a segmented p-type leg as a function of the electrical current (*I*) and also determine the optimal steady-state currents for TEMCs with constant and variable cross-sectional semiconductor areas for each of the proposed geometric models, i.e., the optimum electrical current for the maximum coefficient of performance ($I_{opt}^{COP}$) and the optimum electrical current for maximum cooling power ($I_{opt}^{Q_{c}}$). In the calculations, we consider homogeneous systems with the same material in both segments of the p-type leg and hybrid systems with different materials in each segment of the p-type leg. We analyze two types of hybrid systems in this work, namely direct systems using ${\left(Bi_{0.5}Sb_{0.5}\right)}_{2}Te_{3}$ for the first segment and $Bi_{2}Te_{3}$ for the second segment and inverse systems using $Bi_{2}Te_{3}$ for the first segment and ${\left(Bi_{0.5}Sb_{0.5}\right)}_{2}Te_{3}$ for the second segment. Hybrid systems are considered because is necessary to determine which of the two materials leads to more efficient operation (according to its thermoelectric properties) for a set temperature gradient, to allow our TEC system to be improved and compared with homogeneous systems to evaluate which thermoelectric device shows the highest performance.

#### 4.1.1. Legs with Rectangular Prism Geometry

**Figure 4:**
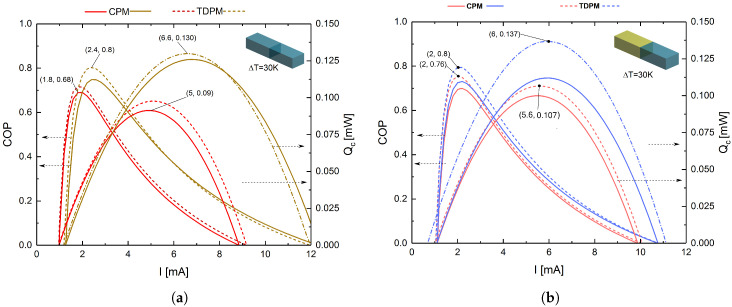
COP and $Q_{c}$ as functions of *I* for legs with the rectangular prism geometry. (**a**) Homogeneous systems based on ${\left(Bi_{0.5}Sb_{0.5}\right)}_{2}Te_{3}$ (red lines) and $Bi_{2}Te_{3}$ (brown lines); (**b**) direct hybrid system based on ${\left(Bi_{0.5}Sb_{0.5}\right)}_{2}Te_{3}$-$Bi_{2}Te_{3}$ (red lines) and inverse hybrid system based on $Bi_{2}Te_{3}$-${\left(Bi_{0.5}Sb_{0.5}\right)}_{2}Te_{3}$ (blue lines). In all cases, the solid and dashed lines correspond to the CPM and TDPM models, respectively.

Figure 4a,b shows the COP and $Q_{c}$, respectively, for legs with the rectangular prism geometry as functions of the electrical current (*I*) at an imposed temperature gradient of ΔT = $T_{h}-T_{c}$ = 30 K, with a value of $T_{c}=270$ K, and the warm-side temperature has been fixed at room temperature $T_{h}=300$ K using a cross-sectional area value of *A* = 25 μm${}^{2}$ and an element length of *L* = 10 μm for each segment; these values have been used in experimental studies of thermoelectric micro-coolers to development low-cost devices [33]. This module was defined as the “original” model, since thermoelectric modules are typically fabricated using legs with rectangular prism geometry, and the other modules were modeled as trapezoidal prisms. It is clear from Figure 4a that the use of $Bi_{2}Te_{3}$ led to a superior performance for the homogeneous system compared with ${\left(Bi_{0.5}Sb_{0.5}\right)}_{2}Te_{3}$, with improvements in the COP and $Q_{c}$ of 12.68% and 39.78%, respectively (the Thomson effect was taken into account in both cases). Figure 4b shows COP and $Q_{c}$ values for the direct hybrid system (${\left(Bi_{0.5}Sb_{0.5}\right)}_{2}Te_{3}$-$Bi_{2}Te_{3}$) and inverse hybrid system ($Bi_{2}Te_{3}$-${\left(Bi_{0.5}Sb_{0.5}\right)}_{2}Te_{3}$). Notice that the inverse hybrid system was found to exhibit higher performance than the direct system, with improvements in the COP and $Q_{c}$ of 6.67% and 29.25%, respectively. For this geometric model, the best configuration for obtaining an optimized TEMC was found to be the inverse hybrid system, which exhibited $COP_{max}=0.8$ and $Q_{c,max}=0.137$ mW at $I_{opt}^{COP}=2$ mA and $I_{opt}^{Q_{c}}=6$ mA, respectively. Thus, the material with the best performance ($Bi_{2}Te_{3}$) should be placed in the first segment of the p-type leg in order to improve the overall performance of the system, because material $Bi_{2}Te_{3}$ is able to absorb more heat and material ${\left(Bi_{0.5}Sb_{0.5}\right)}_{2}Te_{3}$ is able to release a good amount of heat due to the Thomson effect; this phenomena has been previously studied [34,35].

#### 4.1.2. Legs with Trapezoidal Prism Geometry with Small Junction Contact Area

**Figure 5:**
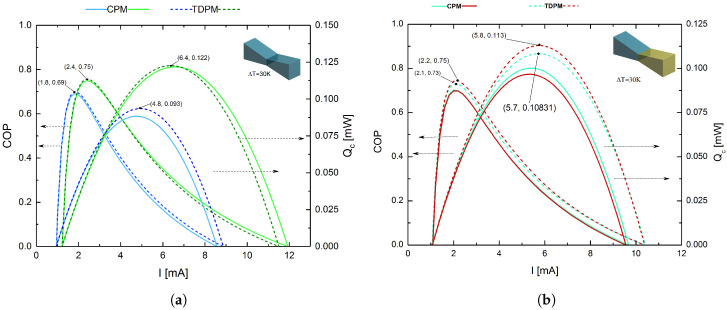
COP and $Q_{c}$ as functions of *I* for legs with the trapezoidal prism geometry with a small junction contact area. (**a**) Homogeneous systems based on ${\left(Bi_{0.5}Sb_{0.5}\right)}_{2}Te_{3}$ (blue lines) and $Bi_{2}Te_{3}$ (green lines); (**b**) direct hybrid system based on ${\left(Bi_{0.5}Sb_{0.5}\right)}_{2}Te_{3}$-$Bi_{2}Te_{3}$ (blue lines) and inverse hybrid system based on $Bi_{2}Te_{3}$-${\left(Bi_{0.5}Sb_{0.5}\right)}_{2}Te_{3}$ (red lines). In all cases, the solid and dashed lines correspond to the CPM and TDPM models, respectively.

Figure 5a,b shows the COP and $Q_{c}$, respectively, for legs with the trapezoidal prism geometry with a small junction contact area as a function of *I*, with ΔT = 30 K, *A* = 25 μm${}^{2}$ at the ends of the leg, and *L* = 10 μm. It can be seen from Figure 5a that the use of $Bi_{2}Te_{3}$ afforded a higher performance for the homogeneous system than ${\left(Bi_{0.5}Sb_{0.5}\right)}_{2}Te_{3}$, as expected from the results discussed above. Again, as shown in Figure 5b, the inverse system exhibited superior performance compared with the direct system, with improvements in the COP and $Q_{c}$ of 7.14% and 13.00%, respectively. The best configuration for this geometric model was found to be the inverse hybrid system, which afforded maximum values of $COP_{max}=0.75$ and $Q_{c,max}=0.113$ mW at $I_{opt}^{COP}=2.2$ mA and $I_{opt}^{Q_{c}}\;=\;5.8$ mA, respectively.

#### 4.1.3. Legs with Trapezoidal Prism Geometry with Large Junction Contact Area

**Figure 6:**
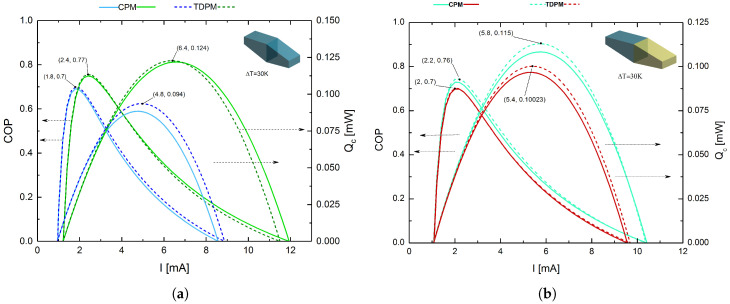
COP and $Q_{c}$ as functions of *I* for legs with the trapezoidal prism geometry with a large junction contact area. (**a**) Homogeneous systems based on ${\left(Bi_{0.5}Sb_{0.5}\right)}_{2}Te_{3}$ (blue lines) and $Bi_{2}Te_{3}$ (green lines); (**b**) direct hybrid system based on ${\left(Bi_{0.5}Sb_{0.5}\right)}_{2}Te_{3}$-$Bi_{2}Te_{3}$ (blue lines) and inverse hybrid system based on $Bi_{2}Te_{3}$-${\left(Bi_{0.5}Sb_{0.5}\right)}_{2}Te_{3}$ (red lines). In all cases, the solid and dashed lines correspond to the CPM and TDPM models, respectively.

Figure 6a,b shows the COP and $Q_{c}$, respectively, for legs with the trapezoidal prism geometry with a large junction contact area as a function of *I*, with ΔT = 30 K, *A* = 25 μm${}^{2}$ at the junction of the segments, and *L* = 10 μm. For the homogeneous system, the use of $Bi_{2}Te_{3}$ once again led to superior performance compared with ${\left(Bi_{0.5}Sb_{0.5}\right)}_{2}Te_{3}$. This result was to be expected, since the COP and $Q_{c}$ of the legs were the same for all models. The main difference observed for this geometric model compared with the previously-discussed system was that the larger junction contact led to the direct system being the best configuration for achieving higher values of COP and $Q_{c}$, affording maximum values of $COP_{max}=0.76$ and $Q_{c,max}=0.115$ mW at $I_{opt}^{COP}=2.2$ mA and $I_{opt}^{Q_{c}}=5.8$ mA, respectively. A comparison of the two trapezoidal prism cases revealed that the model with the larger junction contact area exhibited higher performance, with improvements in the COP and $Q_{c}$ of 1.3% and 1.77%, respectively.

The various leg geometries were modeled with equal lengths and cross-sectional areas (*A* = 25 μm${}^{2}$). For the trapezoidal prism with the small junction contact area, this value was used for the hot and cold sides (i.e., the ends of the leg), whereas for the trapezoidal prism with the large junction contact area, this value was used for the junction of the two segments. The influence of leg geometry on COP and $Q_{c}$ was notable. The results demonstrated that changing the geometry of a segmented leg can significantly decrease or increase its performance, and the inverse hybrid system with rectangular prism geometry was found to exhibit the best cooling power. From Figure 4a,b, which considers the Thomson effect, the STEMC system was found to deliver a maximum cooling power that was 5.10% higher than that of the single-material system. As is demonstrated in this section, trapezoidal leg geometries do not increase cooling power; on the contrary, it decreases because the asymmetric thermal resistance is not sufficient for the Joule heat conduction. In contrast to thermoelectric legs with a conventional geometry, trapezoidal legs could help to lower the overall thermal conductance of the device so as to increase the temperature gradient in the legs, as well as allowing the Thomson effect to be harnessed, an aspect that is generally neglected in conventional rectangular thermoelectric legs. Previous studies have already demonstrated this point for TEG devices, as mentioned in Section 1. Curiously, this does not seem to be the case for TEC systems; on the contrary, the rectangular system proved to be a superior option in terms of thermoelectric performance. Rectangular prism legs may also be preferred over the other leg geometries owing to their efficiency levels and ease of fabrication. In the fabrication of thermoelectric devices, the thermoelectric legs with the desired dimensions are typically obtained by dicing from plated p- and n-type semiconductor wafers using regular cutting saws or electrostatic discharge machining. As such, minimizing the complexity of the manufacturing process might be considered as another concern when considering the optimum leg geometry.

### 4.2. Spatial Temperature Distribution: Thomson Heat Contributions

**Figure 7:**
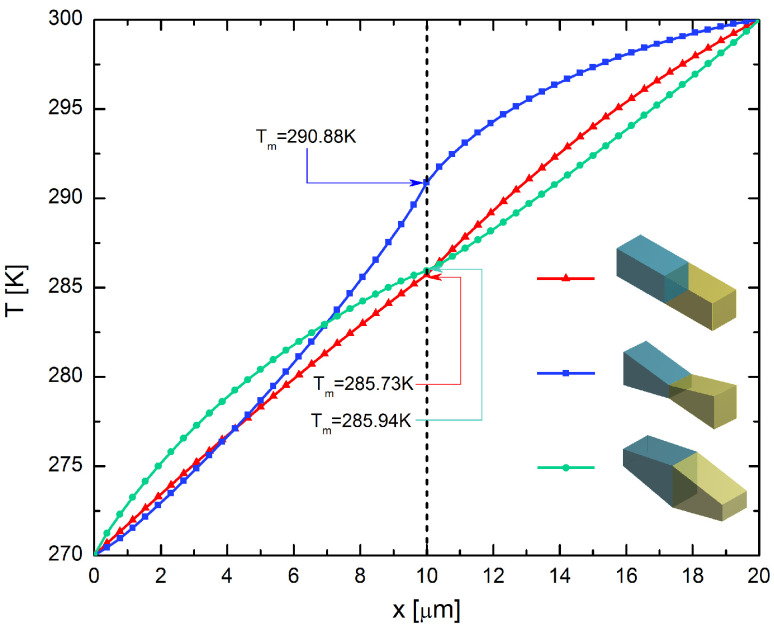
Temperature distributions of the segmented p-type elements with the different geometric models.

In this section, the spatial temperature distribution along the segmented element is analyzed for the TDPM models. A temperature difference of ΔT = 30 K was used for all of the geometric models of the segmented p-type legs considered in this study. The temperature distribution was evaluated when the temperature in the segments had reached a steady-state distribution. Figure 7 shows the spatial temperature distributions for the hybrid systems discussed in the previous section. It is readily apparent that the three systems exhibited very distinct temperature distributions. As discussed above, the highest performance observed in this study was obtained for the inverse hybrid system with rectangular prism geometry. A comparison of the temperature distributions along the three lines revealed that the average temperature in the junction ($T_{m}=285.73$ K) was lower for this system compared with the two trapezoidal prism hybrid systems. In fact, it has been shown that lower values of temperature in the thermocouple act to improve the cooling power, as shown in Figure 7 (red line). It should be noted that the heat absorbed in the first segment is due to $Bi_{2}Te_{3}$, and the heat released in the second segment is due to ${\left(Bi_{0.5}Sb_{0.5}\right)}_{2}Te_{3}$. Compared with the rectangular model, the trapezoidal leg model exhibited two additional features. Firstly, the trapezoidal cross-sectional area produces an asymmetric thermal resistance, and therefore, Joule heat is preferentially conducted toward the larger cross-sectional area. Consequently, for the trapezoidal prism system with small junction contact area, a lower temperature was observed in the first segment and a higher temperature in the second segment, leading to a higher $T_{m}$. The opposite was observed for the trapezoidal prism system with a large junction contact area, leading to a lower $T_{m}$. Secondly, a greater amount of Joule heat is produced in the leg where the cross-sectional area is smaller.

### 4.3. Geometric Optimization of the Rectangular Prism

**Figure 8:**
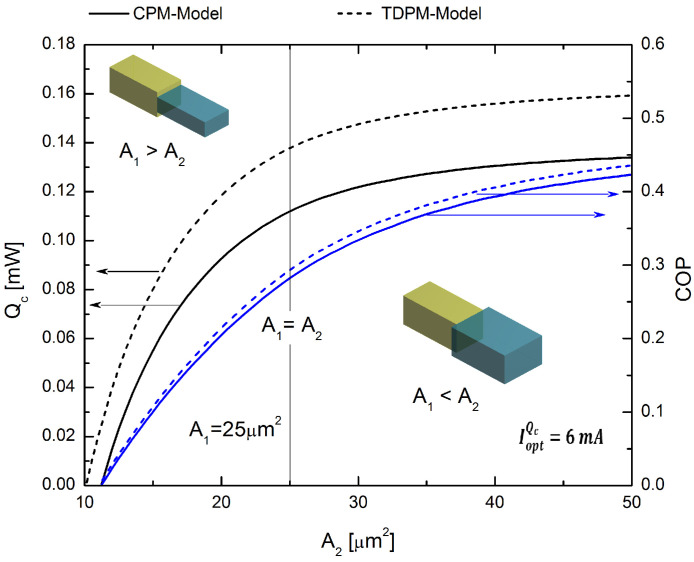
COP and $Q_{c}$ values for the rectangular prism model as a function of the cross-sectional area ($A_{2}$). These data were obtained using the optimal electrical current value of $I_{opt}^{Q_{c}}=6$ mA.

In the next section, we optimize the geometry of our TEMC model. When the areas of the p-type elements are not identical, multivariable optimization can be employed to maximize the cooling power and thermal efficiency of a TEMC system. In the results discussed above, it was found that the best geometric model for optimizing our p-type segmented leg system is the inverse hybrid system with rectangular prism geometry. Using the optimum electrical current values for maximum $Q_{c}$ and for maximum COP, namely, COP, $I_{opt}^{Q_{c}}=6$ mA and $I_{opt}^{COP}=2$ mA, respectively, the segmented p-type leg can be considered optimized in terms of the cross-sectional area ratio that must be used in each segment of the leg. To evaluate the performance behavior of the segmented leg, we set $A_{1}$ = 25 μm${}^{2}$ as a constant value, and the results were determined as a function of the cross-sectional area $A_{c2}$ values. In Figure 8, we use the electrical current of $I_{opt}^{Q_{c}}=6$ mA, which optimizes $Q_{c}$, and it was obtained in Section 4.1.1; see Figure 4b. Figure 8 shows the COP and $Q_{c}$ for values of $A_{2}$ ranging from 10–50 μm${}^{2}$ to allow the evaluation of the two possible relationships between the areas, i.e., when $A_{1}>A_{2}$ and $A_{1}<A_{2}$. The results demonstrate that by increasing the value of $A_{2}$ by 20% (i.e., to $A_{2}=30$ μm${}^{2}$), it is possible to optimize our segmented system, leading to improvements in COP and $Q_{c}$ of 16.67% and 8.03%, respectively.

**Figure 9:**
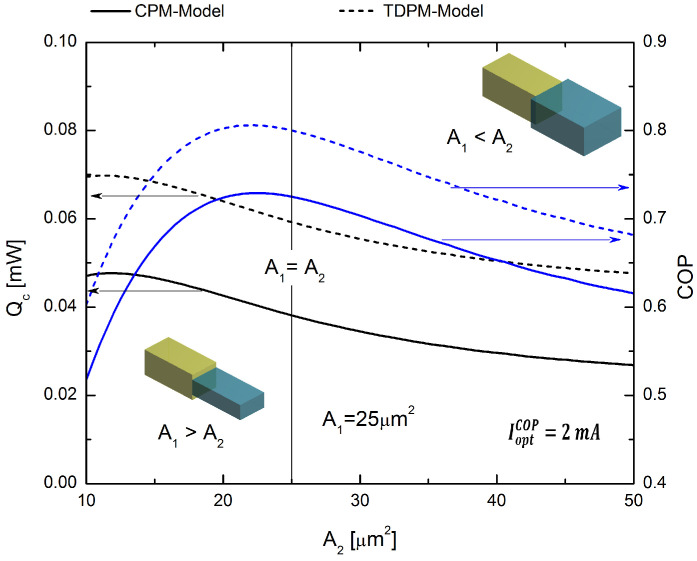
COP and $Q_{c}$ values for the rectangular prism model as a function of the cross-sectional area ($A_{2}$). These data were obtained using the optimal electrical current value of $I_{opt}^{COP}=2$ mA.

Figure 9, calculated using an electrical current of $I_{opt}^{COP}=2$ mA, which optimizes COP obtained in Section 4.1.1 (see Figure 4b), shows the COP and $Q_{c}$ for the same values of $A_{2}$ as used in the above analysis, for the cases when $A_{1}>A_{2}$ and $A_{1}<A_{2}$. The maximum value of COP=0.81 was obtained for the TDPM model, but this led to lower values of $Q_{c}$, as seen in Figure 9, with a maximum value of $Q_{c}=0.07$ mW for $A_{2}$ = 10 μm${}^{2}$, and after this point, $Q_{c}$ decreased. The maximum cooling rate occurs at a certain value of $A_{2}$ = 21.4 μm${}^{2}$ for the TDPM model, and the rate of cooling is lower when the area $A_{2}$ increases. The maximum COP value occurs for the lower area value of $A_{2}$ = 10 μm${}^{2}$, and then, the COP decreases for higher values of $A_{2}$.

This section presents results of the area ratio between both segments, of the p-type leg, on cooling performance of the micro-cooler. The cooling capacity is affected by the Joule and the Peltier effect besides the heat flow conducted from the hot junction to the cold junction where Peltier cooling is counteracted by heat conduction and Joule heating along the TE element. There is, in the system, a competition between these last effects that are related to the electrical and thermal resistivity, which depend on both the length and the cross-sectional area. With an increase in cross-sectional area, the conduction through the thermoelectric element leg increased, or alternatively, due a decrease in thermal resistance of the legs. The variable cross-sectional area makes the thermal resistance asymmetric, and hence, Joule heat is preferentially conducted toward the end with a larger cross-sectional area, referred to as the heat conduction effect. However, more Joule heat is also produced close to the end with the smaller cross-sectional area (referred to as the Joule heat effect here), indicating that the Joule heat effect is dominant over the heat conduction effect. Results show that by increasing the cross-sectional area in Segment 2, it is possible to obtain better cooling power, compared to TE elements having equal cross-sectional area, as we can see in Figure 9a where for higher values of $A_{2}$, better cooling power can be achieved. According to the last statements, trapezoidal legs are not able to achieve enough thermal conductance, as shown in Figure 5 and Figure 6, which achieved lower cooling power values. Previous studies have validated similar results with experimental data; one of these studies integrated a pulse cooler into a small commercial thermoelectric three-stage cooler to operate a laser sensor. For example, Yang and co-workers showed that the thermoelectric microcooler performance can be benefited from the unique shape of the thermoelectric element [36,37]. This finding is parallel with our results.

It has also been reported that the change of the area size in the thermoelectric element might also affect/increase the spreading thermal resistance. The spreading thermal resistance was used in the model of Koh et al. [38,39]. We have checked that the spreading thermal resistance is significant when the area of the first segment is smaller than the second segment ($A_{1}$ > $A_{2}$); while, the spreading thermal resistance is negligible, when the area of second segment is greater than the first segment ($A_{1}$ < $A_{2}$) [37].

## 5. Conclusions

In this study, the influence of the Thomson effect on the performance of a p-type segmented leg in terms of cooling power was evaluated using two semiconductor materials, $Bi_{2}Te_{3}$ and ${\left(Bi_{0.5}Sb_{0.5}\right)}_{2}Te_{3}$, considering the temperature dependence of material properties. We investigated the effects of leg geometry and material configuration on the performance of a TEMC. Three different leg geometries were studied using one-dimensional homogeneous and hybrid systems: rectangular prism, trapezoidal prism with a small junction contact and trapezoidal prism with a large junction contact. The leg configurations were modeled at a steady state with an applied temperature gradient of 30 K for the modeled devices.

The results demonstrated that the hybrid systems (i.e., segmented legs) were capable of delivering higher cooling power ($Q_{c}$) and coefficient of performance (COP) than homogeneous systems, to the extent of 29.25% and 6.67%, respectively, when the Thomson effect is considered. The optimal electrical currents, $I_{opt}^{COP}$ and $I_{opt}^{Q_{c}}$, were determined for all of the proposed geometric models. The inverse hybrid system (i.e., with $Bi_{2}Te_{3}$ and ${\left(Bi_{0.5}Sb_{0.5}\right)}_{2}Te_{3}$ in the first and second segments, respectively) proved to be the optimal configuration of the materials. It should be noted that the configuration of the materials is very important when using materials with different performances for the two segments of the p-type leg. For the devices considered in this study, as $Bi_{2}Te_{3}$ has higher performance values than ${\left(Bi_{0.5}Sb_{0.5}\right)}_{2}Te_{3}$, using $Bi_{2}Te_{3}$ as the first stage helps to improve the overall performance of the system. The temperature distributions in the p-type leg were evaluated for each of the proposed leg geometries, revealing that lower values of temperature in the leg act to improve the cooling power, as in the case of the inverse hybrid system.

The trapezoidal geometries were found to have the opposite effect in the cooling systems than previously reported for power generation systems. Our results shows that rectangular shapes are the best p-type leg geometry to optimize TEMC systems. The asymmetric thermal resistance is not sufficient for the Joule heat conduction in a trapezoidal prism with a large junction contact, even when it is preferentially conducted toward the end with a larger, and therefore, the rectangular model allows greater cooling to be achieved. To optimize the best-performing rectangular prism system, we used the constant value of $A_{1}$ = 25 μm${}^{2}$ and varied the values of $A_{2}$, and the best performance was obtained for $I_{opt}^{Q_{c}}=6$ mA, which increased the values of COP and $Q_{c}$ by 16.67% and 8.03%, respectively.

We demonstrated that for applications requiring high cooling power, hybrid systems with rectangular prism configurations are preferred. In addition, this leg configuration makes it possible to fabricate thermoelectric devices with smaller volumes and lower efficiency losses. The results of this work demonstrate that segmented thermoelectric materials can offer improved efficiency over a wide temperature range and that variation of the length and area of each segment can be used to further optimize the efficiency of the thermoelectric device. Although these improvements are individually small, their effects should be substantial for the series operation of a large number of thermoelectric legs, as is the case in practical applications. Future studies should focus on optimizing the performance of segmented TEMCs according to geometric parameters and by considering the Thomson effect in the calculations.

## Figures and Tables

**Table 1:** Properties of the thermoelectric materials.

Property	Material 1, ${\left({\mathrm{Bi}}_{0.5}{\mathrm{Sb}}_{0.5}\right)}_{2}{\mathrm{Te}}_{3}$	Material 2, ${\mathrm{Bi}}_{2}{\mathrm{Te}}_{3}$	Unit
α	$\left(-62675.0+1610.4T-2.241T^{2}\right)\times 10^{-9}$	$\left(22224.0+930T-0.9905T^{2}\right)\times 10^{-9}$	V K${}^{-1}$
κ	$\left(41214.2-190.7T+0.3285T^{2}\right)\times 10^{-4}$	$\left(62605.0-277.7T+0.4131T^{2}\right)\times 10^{-4}$	W m${}^{-1}$ K${}^{-1}$
σ	$\left(336000-1883.33T+3.2667T^{2}\right)$	$\frac{1}{\left(5112.0+163.4T+0.6279T^{2}\right)\times 10^{-10}}$	(Ωm)${}^{-1}$

## Abbreviation

*A*	Area (m${}^{2}$)
COP	Coefficient of performance
CPM	Constant material property
CPU	Central processing unit
*I*	Electrical current (A)
$j_{\mathrm{el}}$	Electrical current density (A m${}^{-2}$)
$j_{q}$	Heat flux (W m${}^{-2}$)
*L*	Length (m)
$P_{el}$	Electrical power input (W)
$Q_{c}$	Cooling power (W)
$Q_{h}$	Heat flow released (W)
STEMC	Segmented thermoelectric microcooler
T	Temperature (K)
$T_{c}$	Cold side temperature (K)
$T_{h}$	Hot side temperature (K)
$T_{m}$	Temperature in the junction (K)
TDPM	Temperature-dependent material property
TEC	Thermoelectric cooler
TEG	Thermoelectric generator
TEMC	Thermoelectric microcoolers
***Greek letters***
α	Seebeck coefficient (V K${}^{-1}$)
Δ	Difference
κ	Thermal conductivity (W m${}^{-1}$K${}^{-1}$)
μ	Micro
σ	Electrical conductivity [(Ωm)${}^{-1}$]
τ	Thomson coefficient [V K${}^{-1}$]
***Subscripts***
1	appropriate for segment 1
2	appropriate for segment 2
avg	Average values
max	Maximum values
opt	Optimal
***Superscripts***
COP	At maximum coefficient of performance
$Q_{c}$	At maximum cooling power
